# Evaluation of the **constituent** compounds, antioxidant, anticancer, and antimicrobial potential of *Prangos ferulacea* plant extract and its effect on *Listeria monocytogenes* virulence gene expression

**DOI:** 10.3389/fmicb.2023.1202228

**Published:** 2023-07-10

**Authors:** Shahab Jalil Sarghaleh, Behrooz Alizadeh Behbahani, Mohammad Hojjati, Alireza Vasiee, Mohammad Noshad

**Affiliations:** ^1^Department of Food Science and Technology, Faculty of Animal Science and Food Technology, Agricultural Sciences and Natural Resources University of Khuzestan, Mollasani, Iran; ^2^Department of Food Science and Technology, Faculty of Agriculture, Ferdowsi University of Mashhad, Mashhad, Iran

**Keywords:** Jashir, HT 29 cells, *Listeria monocytogenes*, gene expression, confocal laser scanning microscopy

## Abstract

*Prangos ferulacea* plant is very popular in Iran due to its unique properties in treating diseases and its special flavor. To check the characteristics of this plant, first, its extract was extracted using the maceration method. Its chemical composition was investigated using high-performance liquid chromatography (HPLC) that p-coumaric was identified as its main compound, and Fourier-transform infrared spectroscopy (FTIR) showed the presence of functional groups related to phenolic, flavonoid, tannins, and carboxylic acids such as caffeic acid and coumaric acid composition. Total phenol content (TPC), total flavonoid content (TFC), and beta-carotene were equal to 202.04 ± 5.46 mg gallic acid equivalent (GAE)/g dry weight, 1,909.46 ± 13 μg quercetin (QE)/g of dry weight, and 2.91 mg/100 g. The antioxidant property of the extract was evaluated using 2,2-Diphenyl-1-picrylhydrazyl (DPPH) and 2,2′-azino-bis (3-ethylbenzothiazoline-6-sulphonic acid (ABTS) free radical scavenging and ferric reducing antioxidant power assay (FRAP). According to the IC50 obtained for DDPH (274 ± 7.2 μg/mL) and ABTS (120.45 ± 9.6 μg/mL) and FRAP values [1.92 ± 0.05 μg ascorbic acid equivalent (AAE)/g of extract], this extract had high antioxidant properties. Cytotoxicity was evaluated against the survival of HT 29 cells that IC50 was 82.15 ± 0.02 μg/mL. The antimicrobial property of the extract was calculated using disk diffusion agar (DDA), well diffusion agar (WDA), minimum inhibitory concentration (MIC), and minimum bactericidal concentration (MBC). *Listeria monocytogenes* has the highest sensitivity to this extract and inhibition zone based on DDA and WDA method and with an MIC and MBC equal to 16 and 128 mg/mL has the least resistance. The morphology change of *L. monocytogenes* strain was proved through scanning electron microscope (SEM) and confocal laser scanning microscopy (CLSM). The extract caused a significant reduction in the transcription of genes involved in the film formation ability of *L. monocytogenes*. The obtained results fully prove the very practical and pragmatic characteristics of *P. ferulacea*.

## 1. Introduction

In ancient times, in addition to satisfying their nutritional needs, humans also used plants for medicinal purposes, and wild plants in nature made up the majority of these medicinal compounds. After years and in the era of technology, chemical compounds replaced plants, but in recent years, due to some of chemical composition disadvantages, such as the adverse effects on human health caused by the mutagenic and carcinogenic compounds contained in these compounds, the reuse of materials of natural origin has been considered. One of the native plants of Asian regions such as Iran, Iraq, and Turkey and some regions of Europe (Italy) is a medicinal plant from the *Apiaceae* family called *Prangos ferulacea* (Coruh et al., 2007). In Iran, this plant is known as Jashir, and its habitat is the southwestern regions and the slopes of the Zagros mountains (Bazdar et al., 2018). In Turkey and Italy, *P. ferulacea* is used locally for grazing animals, which gives a special taste and smell to its dairy products, and is very popular (Coşkun et al., 2004; Bruno et al., 2021). In the industry, this plant can be used with a dual purpose because it is very aromatic (as a spice and flavoring) and has the properties of an additive (specifically antimicrobial) that can be used as an alternative to synthetic additives (Bazdar et al., 2018).

The functional and biological properties of *P*. *ferulacea* extract are due to its bioactive compounds such as saponin, anthraquinone, tannin, coumarins, linear and angular furocoumarins, monoterpenes (Especially alpha-pinene), sesquiterpenes, phenolics, coumarins, flavonoids, alkaloids, and terpenoids. These compounds are responsible for the antimicrobial, antioxidant, insecticidal, antiviral, anticancer, analgesic, and antidiabetic functions of this plant (Emamghoreishi et al., 2012; Shokoohinia et al., 2014; Badalamenti et al., 2022). The accumulation of free radicals in the human body causes the destruction of normal cells and leads to the occurrence of diseases such as cancer, heart diseases, Alzheimer's, free radical neurological diseases, and premature aging. Therefore, the use of antioxidant compounds to quench and suppress free radicals is necessary. Polyphenols (phenolic acids and flavonoids) in the form of an aromatic ring with hydroxyl groups and alpha-tocopherol as the main antioxidant compounds are in the *P. ferulacea* that quench oxygen-derived free radicals by donating a hydrogen atom or an electron to the free radical. In addition, specifically, coumarin has antiviral and cytotoxic effects, which have been proven in several articles (Musa et al., 2008; Abonyi et al., 2009; Shokoohinia et al., 2014).

Considering the importance of natural plants in the industry and the use of their pragmatic features (Behbahani et al., 2013; Heydari et al., 2020; Alizadeh Behbahani et al., 2021; Falah et al., 2021), *P. ferulacea* plant was investigated in this study. First, *P. ferulacea* extract was extracted with water and its chemical composition was evaluated using high-performance liquid chromatography–mass spectrometry and Fourier-transform infrared spectroscopy (FTIR). In the next step, measuring total phenol content (TPC), total flavonoid content (TFC), beta-carotene, antioxidant properties (DDPH, ABTS, and FRAP of radical scavengers), antimicrobial effect, and cytotoxicity investigation were done. In addition, the structure of *Listeria monocytogenes* bacteria under the effect of the extract was investigated using scanning electron microscopy (SEM) and confocal laser scanning microscopy (CLSM) images. Finally, the effect of the extract on the film-forming genes in *L. monocytogenes* was carried out.

## 2. Materials and methods

### 2.1. Culture medium, strains, and growth conditions

Mueller Hinton agar (MHA), Mueller Hinton broth (MHB), and tryptic soy broth (TSB) were prepared from Difco Laboratories, Detroit, MI, USA. All the strains used in this study were prepared as lyophilized from the National Center for Biological and Genetic Resources of Iran and were, respectively, subcultured in MHB for 24 h at 37°C under sterile conditions. To prepare a fresh microbial suspension, the obtained stock culture was subcultured in slant nutrient agar and washed several times with a sterile ringer solution. A microbial suspension with a 0.5 McFarland standard (1.5 × 10^8^ CFU/mL) was produced by adjusting the suspension's optical density at 630 nm. *L. monocytogenes* strains were cultured in TSB at 37°C for 24 h.

### 2.2. Isolation of *P. ferulacea* extract

The *P. ferulacea* plant used in this project has been identified by the herbarium of Khuzestan University of Agricultural Sciences and Natural Resources. The extract was prepared through the maceration method performed by Behbahani et al. (2017). First, the collected plant from the southern region of Iran was separated from its flowers and soil and washed completely using distilled water. Then, it was kept at 37°C until completely dry, after that, was crushed using a laboratory mill (Moulinex, Germany). In a beaker, the dry plant was mixed with distilled water at a ratio of 1 to 10 (w/v) and the sample was placed on a stirrer at ambient temperature for 24 h with stirring. Then, the sample was filtered using filter paper (Whatman No. 2) and immediately centrifuged. Finally, the evaporation operation was performed using a rotary, and the obtained sample was stored in the refrigerator for later use (Behbahani et al., 2017).

### 2.3. Identification of the chemical structure of the extract

#### 2.3.1. HPLC

The phenolic acid, flavonol, and flavonoid components of plant crude extract were identified and quantified by obtained retention time and standard cure of internal standard (0.1–10 mg/L) through HPLC with the following specifications: a binary pump (G1312A; Agilent 1100); an autosampler (G1330B); a mass spectrometer with an electrospray ionizer source (MS; ESI-; Micromass Quattro Micro; Waters, Milford, MA, USA); reversed phase, a Kinetex C18 column (100 × 2.00 mm; 2.6 μm); capillary voltage, 3.0 kV; cone voltage, 20 V; extractor, 2 V; source temperature of 100°C; desolvation temperature of 350°C; cone gas flow of 30 L/h; desolvation gas flow of 350 L/h; mobile phase [0.1% formic acid (A) and acetonitrile (B)] with a gradient of 0–2 min, 10% B; 2–20 min, 10–60% B; 20–21 min, 60–80% B; 21–25 min, 80% B; 25–26 min, 80–10% B; 26–30 min, 10% B (injection volume of 10 μL and flow rate of 0.300 mL/min) (Terpinc et al., 2016).

#### 2.3.2. FTIR test

In this analysis, the extract was mixed with potassium bromide and then compressed into a suitable tablet form. Then, the FTIR spectrum of the extract was recorded using an FTIR spectrophotometer (Perkin Elmer, USA) in the range of 400–4,000 cm^−1^ wave number with a resolution of 4 cm^−1^ (Behbahani et al., 2019).

### 2.4. Measurement of TPC and TFC

Behbahani et al. (2017) procedures were used to evaluate TPC and TFC. For TPC, first, a concentration of 1% of *P. ferulacea* extract was prepared and mixed with 2 mL distillated water, and 10 μL of this extract was combined with 50 μL of Folin–Ciocalteu reagent and stirred for 3 min. After adding 300 μL of sodium bicarbonate, the solution was shaken for 2 h and the absorption value of the solutions was obtained at a wavelength of 765 nm through a spectrophotometer (Sigma3–30k). Gallic acid solutions (concentrations of 0, 25, 75, 50, 100, 150, 125, 175, and 200 mg of gallic acid/L) were used as a standard. Through the calibration curve of the gallic acid solution and the absorbances obtained from the *P. ferulacea* extract, the TPC value was obtained in terms of mg of GAE/g of extract. For TFC, the response was calculated as mg quercetin equivalence/g of extract, and the aluminum chloride method was used to calculate TFC (Behbahani et al., 2017).

### 2.5. Beta-carotene measurement

The method of Zengru and Tongming (1998) was used based on HPLC {Knauer, Germany, a C18 column [4.6 mm ID × 150 mm (5 μm)], with a UV detector (at 350 nm)} for beta-carotene measurement (Zengru and Tongming, 1998).

### 2.6. Antioxidant property assessment

#### 2.6.1. DPPH assay

DPPH was used to calculate the antioxidant activity through the inhibitory effect of *P. ferulacea* extract against free radicals. First, the 500 to 10 μg/mL concentrations of extracts were prepared in methanol and 1 mL of DPPH solution (0.2 mM, methanolic solution) was added to it and placed in the dark for 30 min at a temperature of 24°C. A wavelength of 517 nm was used to obtain the absorbance of the sample (A sample), and the sample without extract was used as a control (A blank). Finally, antioxidant activity was measured using the following equation: I% = (*A*_blank_ – *A*_sample_/*A*_blank_) × 100.

The obtained number was reported as IC50, which indicates the concentration of the extract that can inhibit 50% of DPPH radicals. The positive control used in this method was vitamin C and TBHQ (Yeganegi et al., 2018).

#### 2.6.2. ABTS inhibition method

ABTS radical scavenging activity was measured according to Labiad et al. (2017) method as follows: preparation of stock solutions of 7 mM ABTS and 2.4 mM potassium persulfate in a 1:1 ratio, kept in the dark at 24°C for 14 h, diluting the solution with ethanol until absorbance of 0.700 ± 0.02 at 734 nm, mixing 2 mL of the resulting inhibitor solution with 200 mL of plant extracts (different concentrations), keeping at room temperature for 30 min to perform the reaction, and stirring the sample and absorbance read at 734 nm. At the same time, the above steps were performed for ascorbic acid (oxo-3-golofuranolactone acid) and TBHQ with different concentrations (1–100 μg/mL) as positive controls. The obtained number was reported in terms of IC50 (Labiad et al., 2017).

#### 2.6.3. The ferric ion (Fe3+) reducing antioxidant power

This procedure is based on the effect of the extract in reducing the ferricyanide complex to ferrous form. First, solutions including extract solution (different nutrients), 2.5 mL of phosphate buffer (0.2 M, pH 6.6), and 2.5 mL of potassium ferricyanide (1% w/v and 2) were prepared, and the incubation process was carried out at 50°C for 20 min. In the step after adding 2.5 mL of trichloroacetic acid (10% w/v), the centrifuge was directed at 1,000 g for 10 min. Absorbance of a solution containing 5 mL of supernatant with 5 mL of deionized water and 1 mL of ferric chloride (0.1%, w/v) was calculated at 700 nm during the reaction time of 30 min. The reducing power of the extracts was expressed as mg ascorbic acid (AA) equivalent per g of dry weight extract (AA/g). All steps mentioned for the extract were performed separately for ascorbic acid as a standard (Labiad et al., 2017).

### 2.7. Cytotoxicity by MTT assay

Cytotoxicity against HT29 cell line (IBRC cell number C10097, National Center for Genetic Resources and Bioscience of Iran) was evaluated through MTT protocol (3-(4,5-dimethylthiazol-2-yl)-2,5-diphenyltetrazolium bromide). The cells were cultured in DMEM_2_ (Dulbecco's Modified Eagle Medium) containing 10% fetal bovine serum and penicillin/streptomycin. The solution was incubated in an incubator with a temperature of 37°C, 95% humidity, and 5% carbon dioxide. Approximately 100,000 cells per one of the wells of 96 houses was added and, also, in the DMEM culture medium, 200 μL of fetal bovine serum and different doses of plant extract (0, 10, 25, 50, 100, and 200 mg/mL) was added to each of the wells. After 24 h of incubation, for cell proliferation measurement, 30 μL of MTT solution (concentration of 5 mg/mL) was added to each of the wells and the plates were placed in a carbon dioxide incubator for 3 h. In the next step, the medium was isolated, 200 μL of dimethyl sulfoxide (DMSO) was added to each of the wells, and the absorbance at a wavelength of 570 nm was recorded using an ELISA reader (ELX 808, Bio Tek Instruments, USA). Cell survival curves were drawn using control cells (Samani et al., 2022).

### 2.8. Microbial assay

#### 2.8.1. Disk diffusion agar method

The strains of *L. monocytogenes, Bacillus cereus, Salmonella enterica* serovar Typhimurium, *Staphylococcus aureus, Escherichia coli, Shigella dysentery*, and *Staphylococcus epidermidis* were investigated as pathogens. One of the common methods to evaluate the antimicrobial activity of plant extracts is DDA. First, concentrations of 20, 40, 60, and 80 mg/mL of *P. ferulacea* plant extracts were prepared, and then, a 0.22 μm syringe microfilter was used for their sterilization. In the next steps, the disks were placed in these solutions for 15 min to be completely soaked in the extract. The prepared microbial suspension equivalent to 1.5 × 10^8^ CFU/mL (corresponding to 0.5 McFarland standard) was used to smear a sterile swab during the inoculation step. The plates were then rotated by 60°, and culturing was carried out again to ensure that the medium surface was thoroughly smeared with the respective microorganisms. Then, the disks that had previously been submerged in particular concentrations of the extract became immobile on the medium's surface. Incubation of the culture medium at 37°C was done for 24 h, and the inhibition zone (IZ) (mm) unit was used to express the antimicrobial effect. Ciprofloxacin antibiotic was used as a positive control (Behbahani et al., 2017).

#### 2.8.2. Well diffusion agar

To measure the diameter of the IZ created by the plant extract, the MHA culture medium was prepared and poured into a petri dish; then, some microbial suspension was spread on the MHA medium using an L-shaped spreader. In the next step, several wells with a diameter of 6 mm were created on the surface of the culture medium, and 20 μL of extract with concentrations of 20, 40, 60, and 80 mg/mL were poured into the wells. The cultures were kept in the incubator for 24 h at 37°C, and the diameter of the IZs around the well was measured and expressed in mm (Behbahani et al., 2017).

#### 2.8.3. Determination of minimum inhibitory/bactericidal concentration by broth microdilution method

The MIC measurement was done as follows: preparation of a culture with a number of 1.5 × 10^8^ CFU/mL (equivalent to 0.5 McFarland standard) of bacteria, preparation of extract solution in DMSO solution (1 mg/mL), successive dilution of the solution with MHB, adding 125 μL of microbial suspension to each well of the plate (96-well plate) (equivalent to 0.5 McFarland standard), keeping in an incubator at 37°C for 24 h, and adding 25 μL of reagent solution of triphenyltetrazolium chloride (5 mg/mL). In the wells where the microbe had grown, a deep red or amethystine color appeared in less than half an hour. As a result, the lowest concentration in which no microbial growth was observed and no color change was observed was considered MIC. To determine the MBC value, 100 μL of the media from each well (absence of red color in the plate) were cultured on MHA and incubated at 37°C for 24 h. The minimum dilution that caused whole prevention of growth was considered MBC (Behbahani et al., 2017).

### 2.9. Morphology assessment

#### 2.9.1. SEM

First, a microbial strain of *L. monocytogenes* was separated by centrifuging the microbial suspension (5,000 × g for 5 min), and then washing with 0.1 M sodium phosphate buffer (pH 7) and filtration with a polycarbonate filter were done. The concentration of 2.5% (v/v) glutaraldehyde solution was used for stabilization, and the incubation of the dissolved microbial sample was done at refrigerator temperature for 2 h. Distilled water and ethanol were used for the final dehydration and washing, respectively. After drying the sample in a vacuum, it was covered with a layer of gold and examined SEM (LEO 1450 VP model, Germany) (Alizadeh Behbahani et al., 2020).

#### 2.9.2. CLSM

The method of Bandara et al. (2013) was used to evaluate the effect of the extract on the biofilm creation of *L. monocytogenes*. For this test, biofilm was prepared using pre-sterilized flat bottom six-well plates (Iwaki) and pre-sterilized plastic coverslips (Thermanox plastic coverslips; Nalge Nunc International, Rochester, NY, USA). The *L. monocytogenes* suspension was added to the first plate (pre-sterilized coupons (stainless steel, Ø 12.7 mm) were placed in the wells of three different six-well plates) and incubated for 24 h in an orbital shaker (75 rpm) at 37°C. The pre-washed coupons were stained with live and dead spots and examined (Bandara et al., 2013).

### 2.10. Investigating the effect of the extract on the gene expression of biofilm formation by *L. monocytogenes*

#### 2.10.1. RNA isolation and cDNA synthesis

First, *L. monocytogenes* bacteria were cultured in TSB medium at 37°C, and to achieve a bacterial count of 10^8^ CFU/mL, 2 mL of the culture was added to 40 mL of fresh TSB by measuring through a spectrophotometer and achieving an OD of 600 nm. It was diluted equal to 0.3. The 4.8 mL aliquot of the bacterial sample was mixed with 200 μL of pennibacterin solution (adding PBS for dilution) to prepare dilutions of 1.7, 3.4, and 6.8 μg/mL (diluted culture mixture with PBS as a positive control). Then, the obtained compounds were kept in an incubator at a temperature of 37°C for 4 h with stirring. After centrifugation, washing the obtained sample with PBS, extracting the total RNA from the washed sample using a commercial kit (RNAiso Plus kit; TaKaRa Dalian Biotechnology, Dalian, China) was done. Then, RNA was analyzed using agarose gel electrophoresis (DYY-8C, Beijing Liuyi Biotechnology, Beijing, China), and RNA purity was analyzed by spectrophotometer (MD2000D, Biofuture, Cambridge, UK) (Li et al., 2018).

For cDNA synthesis, first purification and removal of residual DNA with RNase-Free DNase (RQ1; Promega Biotech, Wisconsin, USA) were performed. Then, the following steps were carried out in order: Incubation of 10 μL of the mixture including 1 μL 10 × reaction buffer, 1 μL DNase, 7 μL RNase-Free H_2_O, and 1 μL extracted RNA at 37°C for half an hour, stopping the reaction with 1 μL of DNase stop solution, incubation of the mixture at 65°C for 10 min, using a commercial reaction kit (PrimeScript™ RT reagent Kit; TaKaRa Dalian Biotechnology, Dalian, China) for cDNA synthesis, incubation of 20 μL of a mixture consisting of 1 μL reverse transcriptase (RT) mix, 2 μL RT primer mix, 4 μL 5 × buffer, 2 μL RNase-Free dH_2_O, and 11 μL DNA-free RNA mix at 37°C for 15 min, heating the mixture for 5 s in 85°C to inactivate the enzyme (Li et al., 2018).

#### 2.10.2. Real-time polymerase chain reaction

The results of RT-PCR were calculated using the 2^−Δ*ΔCq*^ method described by Kim et al. (2021). RT-PCR was applied to appraise the expression of biofilm-related and reference genes (Miao et al., 2019). Table 1 contains a list of the RT-PCR primer sequences. RT-PCR was performed in a 25-μL system using SYBR^®^ Premix Ex Taq™ II (TakaRa). One cycle of 95°C for 30 s, 40 cycles of 95°C for 5 s and 60°C for 30 s, and dissociation steps of 95°C for 15 s and 60°C for 30 s were all included in the cycling conditions.

**Table 1:** Genes used in this study.

**Gene**	**Primer**
*sigB*	Forward GATGATGGATTTGAACGTGTGAA
	Reverse CGCTCATCTAAAACAGGGAGAAC
*agrA*	Forward ATGAAGCAAGCGGAAGAAC
	Reverse TACGACCTGTGACAACGATAAA
*prfA*	Forward CGGGAAGCTTGGCTCTATTTG
	Reverse GCTAACAGCTGAGCTATGTGC
*hly*	Forward AACCAGATGTTCTCCCTGTA
	Reverse CACTGTAAGCCATTTCGTCA
*plcB*	Forward CAGGCTACCACTGTGCATATGAA
	Reverse CCATGTCTTCYGTTGCTTGATAATTG
*flaA*	Forward CTGGTATGAGTCGCCTTAG
	Reverse CATTTGCGGTGTTTGGTTTG
*inlB*	Forward AAGCAMGATTTCATGGGAGAGT
	Reverse TTACCGTTCCATCAACATCATAACTT

### 2.11. Statistical analysis

Experiments were repeated three times, and data were analyzed by using a one-way analysis of variance (ANOVA) (SPSS, version 17, SPSS Inc., Chicago, IL). Means were further classified using the Tukey as a post-test. *P*-values of 5% were considered significant.

## 3. Results and discussion

### 3.1. Chemical properties

#### 3.1.1. Phenolic profile

The compounds in the extract were identified based on the comparison of the retention time of the standard samples with the extract sample and their amount were obtained based on the curve peak areas (Table 2). As can be seen in Table 2, according to the retention time, catechin (4.31 min), rutin (7.21 min), p-coumaric acid (10.18 min), myricetin (11.59 min), caffeic acid (14.22 min), luteolin (14.45 min), and kaempferol (18.11 min) were detected. P-coumaric compounds with the amount of 650 μg/g dry weight (DW) and kaempferol with the amount of 256.2 μg/g DW were the highest and lowest phenolic components of the extract, respectively. Due to the polar nature of phenolic compounds and flavonoids, their extraction through water or methanol will result in high extraction efficiency of these compounds, which is in accordance with the results obtained in various articles (Abdul Razak et al., 2012; Abarca-Vargas et al., 2016). The presence of phytochemical compounds as secondary metabolites such as coumarin derivatives in the extracts of various species of prangos plant has been reported in different studies (Meshkatalsadat and Mirzaei, 2007; Badalamenti et al., 2022). In addition, quercetin and myristin derivatives have been identified as glycosidic flavonoids in *Prangos uechtritzii* (Schieber et al., 2002; Mosić et al., 2019). In the study by Dall'Acqua et al. (2022), who identified the bioactive compounds in prangos species by HPLC, the obtained secondary metabolites were grouped into concentrated tannins, hydrolyzable tannins, coumarins, and flavonoid derivatives (Dall'Acqua et al., 2022). Similar to the results of this study, the presence of phytochemical compounds of coumarins (as the dominant compound) and some phenolics such as caffeic acid and quercetin has been reported in various species of the prangos family (Delnavazi et al., 2016; Zengin et al., 2020; Albayrak et al., 2021; Bruno et al., 2021). In addition, in the study conducted by Terpinc et al. (2016), rutin was identified as the main phenolic compound in buckwheat with a value of 92 ± 1 μg/g DW (Terpinc et al., 2016).

**Table 2:** Specifications of peaks obtained in HPLC of *Prangos ferulacea* crude extract.

**Peak IDs**	**RT (min)**	**Peak area (%)**	**Recovery (%)**	**Concentration (μg/g)**
Catechin	4.31	10.04875	95	305.4
Rutin	7.21	11.18744	97	263.6
P-Cumaric acid	10.18	28.59037	98	650.9
Myricetin	11.59	13.30039	92	301.4
Caffeic acid	14.22	18.4662	93	488.2
Luteolin	14.45	8.808	89	261.8
Kaempferol	18.11	9.5980	94	257.2

#### 3.1.2. Identification of *P. ferulacea* extract by FTIR

**Figure 1:**
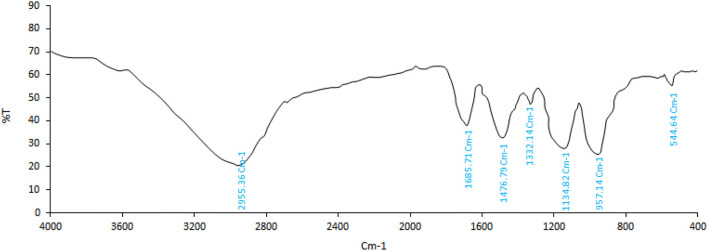
FTIR spectrum of *Prangos ferulacea extract*.

FTIR analysis is used to identify bonds and functional groups in the structure of various chemical compounds. With the simplest and fastest sample-handling techniques, the FTIR spectrum provides a precise representation of all chemical bonds present in the cells (Machana et al., 2012). The structural/chemical characterization provided by IR spectroscopy based on functional group vibrations can be used to elucidate the organic structure. A fingerprint IR spectrum can be used to identify unknown compounds, while characteristic absorption bands can be used for compound-specific detection or to confirm quality markers in plant extracts or distillates (Agatonovic-Kustrin and Morton, 2020). The spectrum of the *P. ferulacea* extract sample is presented in Figure 1. As can be seen from Figure 1, the CH_2_ groups stretching vibrations of alkanes are determined at a peak of 541 cm^−1^. The presence of a peak in the wavenumber of 1,000–1,332 cm^−1^ indicates C-O bonds related to alcohols, carboxylic acids, esters, and ethers. The signal of 1,476 cm^−1^ describes C=C stretching vibrations of the aromatic ring (Albayrak et al., 2021). In addition, the peak of 1,685 cm^−1^ was ascribed to the C=O vibrations related to aldehyde groups. The C-H stretching vibrations that are often related to the alkaline compound in the extract were placed at 2,955 cm^−1^ (Alizadeh Behbahani et al., 2020). The peaks in the region of 3,000–3,500 cm^−1^ are related to the stretching vibrations of hydroxyl (O-H) which may be related to alcohol groups or carboxylic acids (Samani et al., 2022). The presence of phenolic compounds, flavonoids, carboxylic acids such as caffeic acid and coumaric acid, as well as aromatic compounds is probable according to the peaks formed.

### 3.2. TPC and TFC assessment

Gallic acid and quercetin were used as standards to measure TPC and TFC, respectively. Based on the obtained results, the value of 202.04 ± 5.46 mg GAE/g DW as TPC and 1,909.46 ± 13 μg QE/g of DW as TFC were obtained.

The phenolic content 11.75 mg GAE/g extract and flavonoid 2.26 mg QE/g extract of methanolic fruit extract of *P. ferulacea* by Cesur et al. (2017), phenol level 20.25–35.68 mg GAE/g and flavonoid 8.36–56.79 mg RE/g by Zengin et al. (2019), phenol level 44.44 mg GAE/g and flavonoid 13.02 mg RE/g for *P. ferulacea* by Zengin et al. (2020), and total phenolic level 72.33 mg GAE/g and flavonoid 35.31 mg QE/gin the methanolic extract of *Ferulago angulata* was reported by Ghasemi Pirbalouti et al. (2016). The amount of beta-carotene was calculated to be 2.91 mg/100 g. For sources rich in beta-carotene, such as yellow pumpkin, green pepper, *Portulaca oleracia*, field beans, and French beans, the amounts of 1.180, 1.02, 1.7, 0.554, and 0.393 mg/100 g have been reported (Kandlakunta et al., 2008). According to these results, *P. ferulacea* has high amounts of beta-carotene. In addition, the presence of high beta-carotene content in *Apium graveolens* and *Prunus armeniaca L*. was reported and its effects on preventing CC14-induced hepatic steatosis and damage were probably due to its high antioxidant and radical scavenging properties (Ozturk et al., 2009; Khan et al., 2011).

In general, depending on the type of plant, variety, different parts of the plant, and the climatic conditions of the place of cultivation and the extraction method, the amount of phenolic and flavonoid compounds of this plant is very variable (Behbahani et al., 2017). For example, the flower parts of the plant have more flavonoids than the leaves through extraction with water, but in another study, the amount of these compounds through methanolic extraction is more in the leaves than in the flowers (Bazdar et al., 2018), but in general, the aerial parts have higher concentrations of phenols and flavonoids compared to *P. ferulacea* fruits (Zengin et al., 2020). In addition, various enzymes are effective in the synthesis of flavonoid and phenolic compounds (phenolic compounds are responsible for color, smell, and taste). These enzymes increase in the initial growth of plant cells and before full growth, but after maturation and ripening, these enzymes are destroyed, so the production of these compounds should also be reduced (Behbahani et al., 2017).

### 3.3. Antioxidant quality

The human body is always under the influence of various stresses, which cause the production of free radicals and many harmful compounds, so, to overcome these stresses, antioxidants are the best defense mechanism. Therefore, human foods are very important in terms of investigating their antioxidant properties. For this purpose, the antioxidant property of *P. ferulacea* extract was measured by DPPH and ABTS inhibitory activity and FRAP assay.

**Figure 2:**
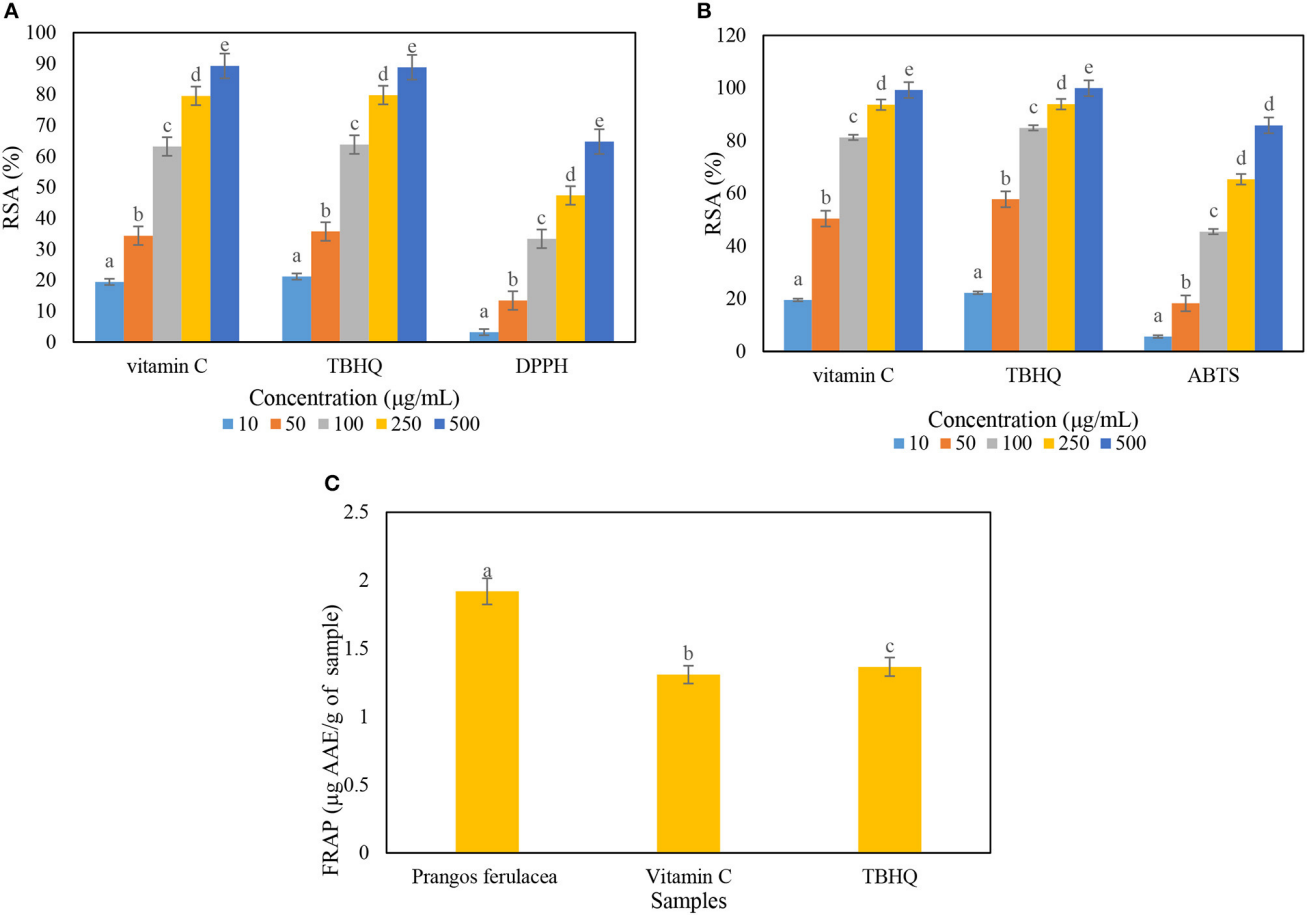
Determination of radical scavenging activity (RSA) percentage of *Prangos ferulacea* extract on **(A)** DPPH, **(B)** ABTS, and **(C)** FRAP radicals. Letters a–e in the **(A, B)** indicate the difference between different concentrations in an antioxidant and in **(C)** a–c indicate difference between different antioxidants.

In DPPH inhibition assay, the amount of antioxidant activity of *P. ferulacea* extract was obtained by examining the power of different concentrations of the extract in inhibiting DPPH radicals, drawing a graph of absorption vs. concentration, and calculating the IC50 value through the slope of the line. In this method, vitamin C and TBHQ were used as reference antioxidant compounds. The IC 50 index is defined as a concentration of antioxidants that can inhibit 50% of radicals. The graph related to the DPPH inhibitory activity of the extract sample was compared with vitamin C (natural antioxidant) and TBHQ (synthetic antioxidant) (Figure 2A). The IC50 values for the extract sample, vitamin C, and TBHQ were 274 ± 7.2, 62.64 ± 3.5, and 59.62 ± 3.4 μg/Ml, respectively, which induces antioxidant activity about 4.5 times less than the positive control samples. Based on the chart obtained from the ABTS test, the value of IC 50 for extract, vitamin C, and TBHQ samples was calculated as 120.45 ± 9.6, 38.05 ± 2.4 and 31.73 ± 1.8 μg/mL, respectively (Figure 2B). The FRAP method is based on the ability of antioxidant compounds to reduce the ferric (Fe^3+^) to the ferrous (Fe^2+^) form, which ascribes the ability of the extract compounds to donate electrons that can react with free radicals and reduce oxidative stress. The extract of *P. ferulacea* showed FRAP value as 1.92 ± 0.05 μg AAE/g of extract and FRAP values of vitamin C and TBHQ were 1.3 ± 0.033 and 1.36 ± 0.026 μg AAE/g of extract, respectively (Figure 2C). The amount of FRAP of the extract sample is only approximately 30% lower than the positive control (vitamin C and TBHQ), which indicates a high antioxidant activity.

The amount of 242 μg/mL for DPPH scavenging IC50 of *P. ferulacea* extract is reported, which is according to the results of the study (Coruh et al., 2007). Similar to the results of this study, Labiad et al. (2017) reported the value of IC50 is equal to 275.71 ± 11.26 μg/mL and 127.38 μg/mL, respectively, for DPPH and ABTS radical scavenging activity of *Thymus satureioides* extract obtained from hexane solvent (Labiad et al., 2017). IC50 value of free radical scavenging power in terms of DPPH and ABTS of *P. ferulacea* essential oil is equal to 726.5 μg/mL (360 times lower than Trolox as a control) and 89.5 μg/mL (about 65 times lower than Trolox), respectively (Bruno et al., 2021). Badalamenti et al. (2022) investigated the antioxidant activity of *P. ferulacea* essential oil extract obtained from flowers and leaves through anti-H_2_O_2_ factors and anti-radical activity of ABTS. They reported an IC50 value of 60 μg/mL related to anti-H_2_O_2_ activity for flowers and an IC50 value of 100 μg/mL for ABTS. They also calculated the anti-H_2_O_2_ activity with IC50 value of 50 μg/ml and the highest anti-radical effect (IC50 value of 500 μg/mL) for ABTS of *P. ferulacea* leaf essential oil (Badalamenti et al., 2022). The main defensive effects of natural antioxidants in natural foods are related to vitamins, phenols, and carotenoids. In the performed classification, ascorbic acid and phenolics are considered hydrophilic antioxidants, and carotenoids are considered lipophilic antioxidants (Labiad et al., 2017). As a result, the higher radical inhibition activity of the hydroalcoholic (hydrophilic) extract can be attributed to the higher amount of hydrogen-donating phenolic antioxidants in the ethanolic-blue (polar) extract compared to the non-polar extract (Kandlakunta et al., 2008; Zengin et al., 2020). The DPPH inhibitory activity and ABTS assay methods are more based on hydrophilic oxidants, but the ABTS method can be used in both organic and aqueous solvent systems compared to other antioxidant methods because it performs better for evaluating the anti-radical capacity of both hydrophilic and lipophilic antioxidants (Bazdar et al., 2018). Williams et al. (2004) and Labiad et al. (2017) stated that phenolic compounds have properties of hydrogen donors and single oxygen quenchers, and their redox potential plays an important role in determining antioxidant potential (Williams et al., 2004; Labiad et al., 2017).

### 3.4. Cytotoxic quality

**Figure 3:**
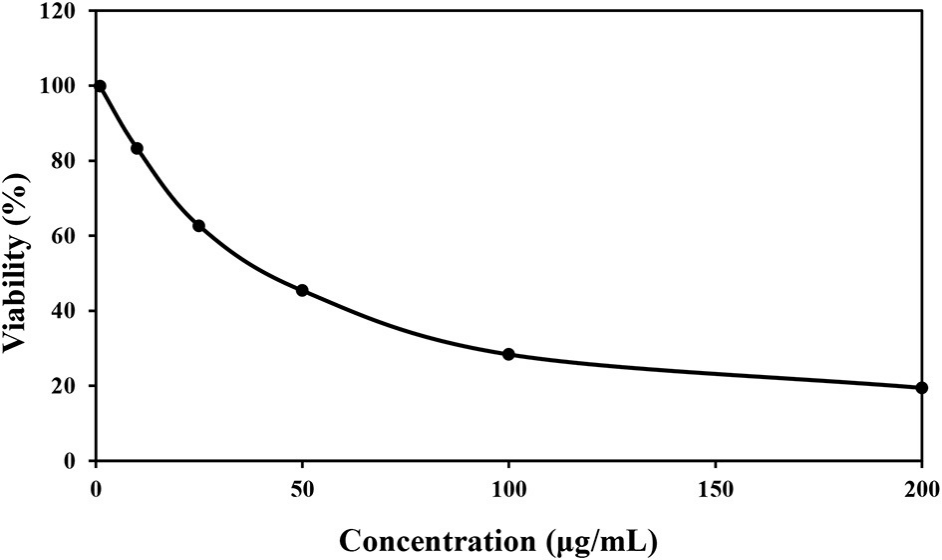
Cytotoxic effect of various concentrations of *Prangos ferulacea* extract on survival of HT29 cell line.

According to the obtained Figure 3, the survival of HT 29 cells in concentrations of 0, 10, 25, 50, 100, and 200 μg/mL was 99.82, 83.28, 62.61, 45.34, 28.36, and 19.43%, respectively, and the value of IC50 was calculated as 82.15 ± 0.02 μg/mL. The lowest percentage of cell viability was observed in the concentration of 200 μg/mL of *P. ferulacea* extract. It can also be seen that with the increase in the concentration of *P. ferulacea* extract, the viability of HT 29 cells decreased remarkably. Bruno et al. (2021) evaluated the cytotoxic activity of *P. ferulacea* essential oil by HCT116 (colon carcinoma), MDA-MB 231 (breast adenocarcinoma), and A375 (melanoma). Their results showed that the essential oil has a moderate cytotoxic activity on the tested cell lines and the IC50 values of MDA-MB 23, A375, and HCT116 cells were 22.41, 25.08, and 30.35 μg/L (Bruno et al., 2021). In several publications, the relationship between antioxidant activity and cytotoxicity has been stated, so that the higher the antioxidant activity, the lower the survival rate of cancer cells (Hou et al., 2007; Rodrigues et al., 2020). Antioxidant compounds reduce the oxidative stress of body cells by absorbing free radicals and reduce the burden of malignancy in body cells. Phenolic compounds show their anticancer effect by preventing metabolic enzymes involved in the activation of carcinogens or stopping the cancer cell cycle (Abdelhady and Badr, 2016).

### 3.5. Antimicrobial properties

**Figure 4:**
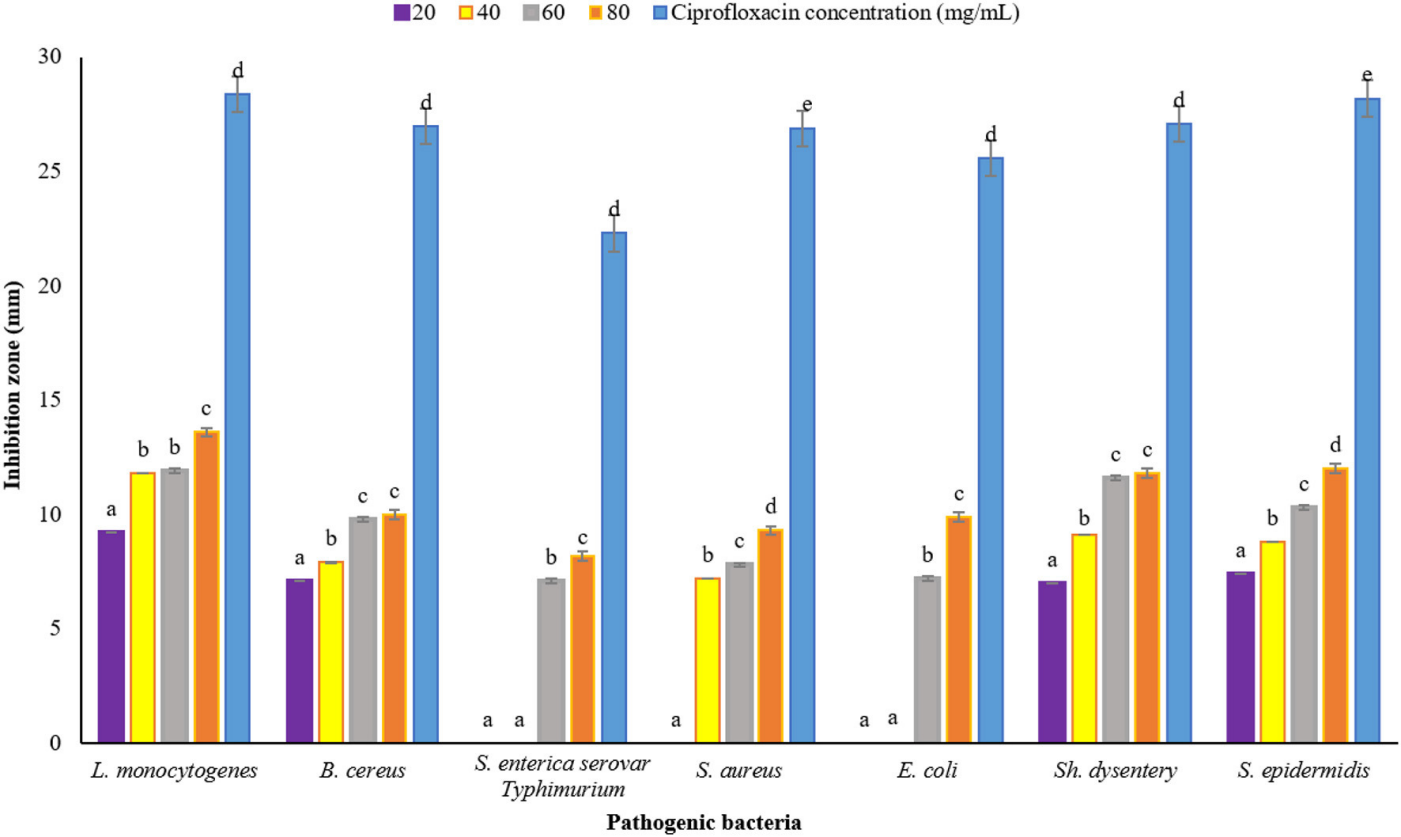
Average IZ (mm) of *Prangos ferulacea* aqueous extract against pathogenic bacteria, based on DDA method [Different letters (a–e) in each strain show significant difference at *p* < 0.05]. Different letters (a–e) in each strain show significant difference at *p* < 0.05.

Plants have been very important as medicine and food (flavor agent) since ancient times. One of the main characteristics of plants is their antimicrobial property, which can be used in the industry to reduce the use of chemical additives that have adverse effects on health. In this study, DDA and WDA methods were used to investigate the antimicrobial properties of *P. ferulacea* extract. The results of investigating the antimicrobial effect of *P. ferulacea* aqueous extract against pathogenic bacteria, based on the DDA method, are given in Figure 4. In all investigated concentrations, the diameter of the IZ of *L. monocytogenes* is significantly higher than other investigated strains. As can be seen from Figure 4, the concentration of 20 mg/mL extract had no effect on *S. enterica* serovar Typhimurium, *E. coli*, and *S. aureus* bacteria, but the concentration of 40 mg/mL had no effect on *S. enterica* serovar Typhimurium and *E. coli* as gram-negative bacteria. *S. enterica* serovar Typhimurium and *E. coli* bacteria at a concentration of 60 mg/L have created an IZ of 7.10 mm and 7.20 mm, respectively, which is the lowest inhibition rate among other strains and these two numbers were not significantly different (*p* < 0.05). At the concentration of 80 mg/mL, significantly (*p* < 0.05) the IZ of *S. enterica* serovar Typhimurium is lower than the others, which indicates the high resistance of this strain to *P. ferulacea* extract. In general, this extract was more effective against gram-positive bacteria than gram-negative bacteria, and high concentrations of the extract significantly (*p* < 0.05) had a greater inhibitory effect. In addition, compared to the antibiotic ciprofloxacin (Figure 4), it is observed that *L. monocytogenes* has the highest sensitivity to this antibiotic, and its IZ under the influence of this antibiotic is almost double the IZ under the influence of the extract. In recent years, due to the indiscriminate use of antibiotics, resistance to them has been created in the human body; as a result, the use of medicinal plant extracts instead of antibiotics is a suitable option to deal with the resistance created against them.

**Figure 5:**
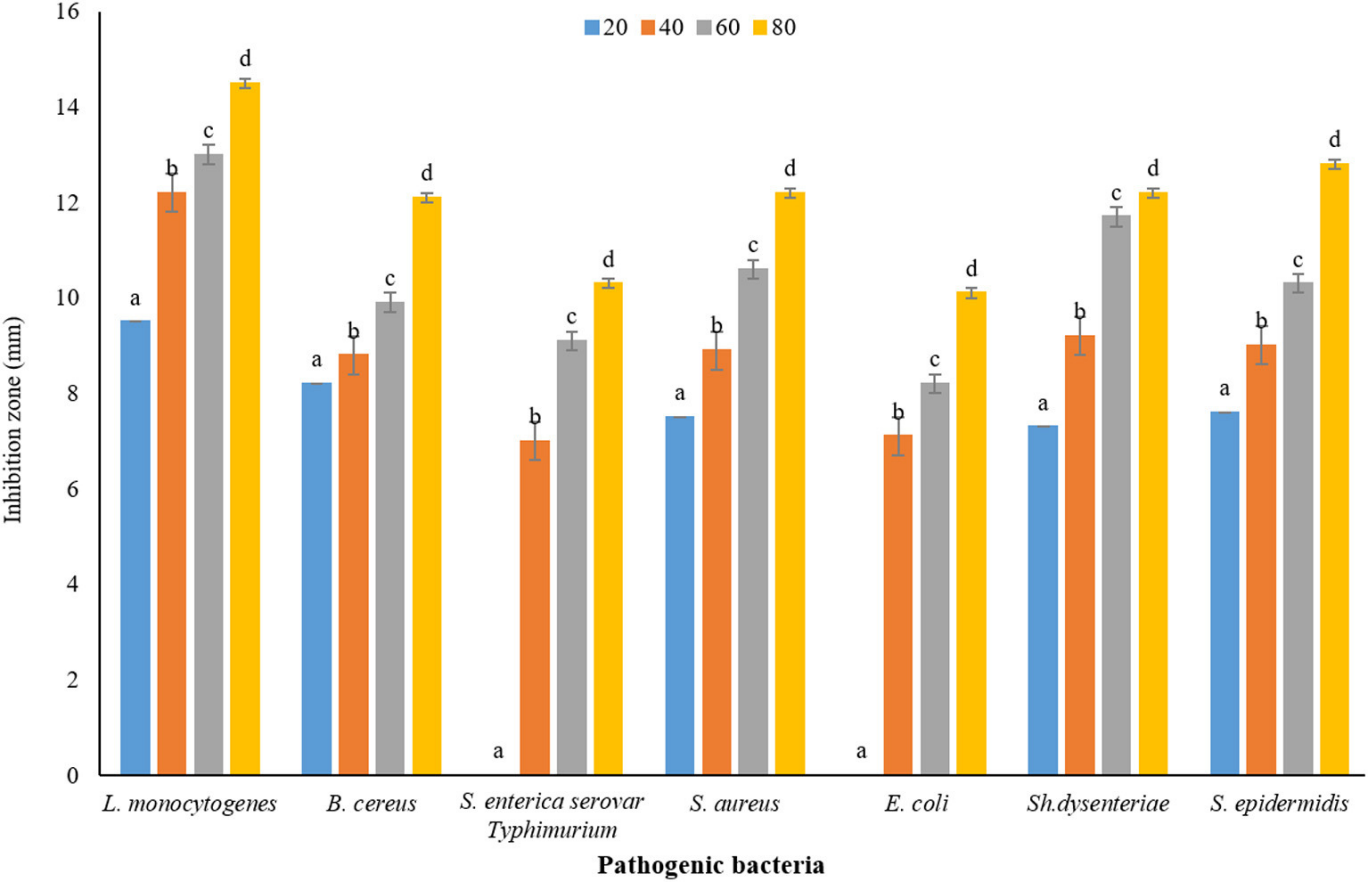
Average IZ (mm) of *Prangos ferulacea* aqueous extract against pathogenic bacteria, based on WDA method [Different letters (a–d) in each strain show significant difference at *p* < 0.05]. Different letters (a–d) in each strain show significant difference at *p* < 0.05.

The results of measuring the average diameter of the IZ (mm) of *P. ferulacea* aqueous extract against pathogenic bacteria, based on the WDA, are presented in Figure 5. According to the results obtained from WDA, in all the investigated concentrations, the maximum diameter of the IZ was created in the culture medium of the pathogen *L. monocytogenes* (20 mg/mL, 9.5 ± 0.16 mm; 40 mg/mL, 12.20 ± 0.37 mm: 60 mg/mL, 13 ± 0.18 mm; and 80 mg/mL, 14.5 ± 0.25 mm). In addition, in general, *E. coli* has shown the highest resistance against *P. ferulacea* aqueous extract. The concentration of 20 mg/mL had no effect on gram-negative pathogenic bacteria *S. enterica* serovar Typhimurium and *E. coli*. Similar to the results of the DDA (Figure 4), with the increase in the concentration of the extract from 20 to 80 mg/mL, the IZ has increased significantly, which indicates a direct relationship between the concentration of the extract and the diameter of the inhibition halo. At a significance level of 5% (*p* < 0.05), the effect of the extract concentration on the IZ is quite significant.

Similar to the results of this study, Behbahani et al. (2017) reported that a low concentration of tarragon extract (5 mg/mL) had no effect on *E. coli* bacteria, and at high concentrations, a non-growth halo was created in the plate of this bacterium. In addition, gram-negative bacteria were more resistant to this extract than gram-positive (Behbahani et al., 2017). In line with the results of this study, the antibacterial effects of four extracts of *P. ferulacea* (hexane, ethanol, methanol, and water) on the bacteria *Micrococcus luteus, S. aureus, B. cereus*, and *B. subtilis* and *S. enterica* serovar Enteritidis*, E. coli, Proteus mirabilis*, and *Klebsiella pneumoniae* have shown that this extract has a strong antimicrobial effect and the least effective against *E. coli* was reported (Durmaz et al., 2006).

The results of MIC and MIB tests of *P. ferulacea* aqueous extract against pathogenic bacteria can be seen in Table 3. According to the obtained results, the MIC order of the studied pathogens is as follows:

**Table 3:** MIC and MBC of the aqueous extract of *Prangos ferulacea* for some pathogenic bacteria.

**Bacteria**	**MIC (mg/mL)**	**MBC (mg/mL)**
*L. monocytogenes*	16	128
*B. cereus*	32	256
*S. enterica* serovar Typhimurium	64	>512
*S. aureus*	32	512
*E. coli*	128	>512
*Sh. dysenteriae*	32	512
*S. epidermidis*	32	256

*E. coli* (128 mg/mL) > *S. enterica* serovar Typhimurium (64 mg/mL) > *B. cereus* = *S. aureus* = *S. epidermidis* = *Sh. dysentery* (32 mg/mL) > *L. monocytogenes* (16 mg/mL).

As can be inferred from Table 3, the MBC factor of *E. coli* and *S. enterica* serovar Typhimurium bacteria with a value >512 mg/mL was higher than other bacteria, and *L. monocytogenes* with an MBC equal to 128 mg/mL was the least resistant to the extract. According to the comparison of the results obtained from the IZ (DDA and WDA methods) with the results of MIC and MBC, it can be concluded that *L. monocytogenes* was the most sensitive and *E. coli* the most resistant strain against *P. ferulacea* aqueous extract. Similar to the results of this study, Dikpinar et al. (2018) explained that *Ferulago trachycarpa* methanol extract had no effect against *S. aureus* and *E. coli* bacterial strains (Dikpinar et al., 2018). Zengin et al. (2020) investigated the antimicrobial properties of seven *Apiaceae* species. Based on the obtained results, they reported MIC and MBC values of 0.75 and 1.5 mg/mL for *P. ferulacea* and generally stated that *Apiaceae* species have moderate antimicrobial activity (Zengin et al., 2020). Since the amount of plant compounds, especially phenolic compounds, varies according to species, cultivation conditions, climate, harvest stage, harvest area, and different parts of the plant, as a result, their antimicrobial activity is completely variable (Alizadeh Behbahani et al., 2020). Various factors affect the antimicrobial activities of the extracts, which include the type and amount of antimicrobial compound (phenolic compounds, quinones, beta-carotene, flavonoids, and tannins), the type of microorganisms, and the way the extract is mixed with the microorganisms. In general, phenolic compounds are the most important antimicrobial compounds in plant extracts, usually their mechanism of action is based on enzymatic inhibition of the oxidized compound or blocking sulfhydryl groups of proteins (Dholwani et al., 2008). The type of cell membrane of gram-positive (lacking lipopolysaccharide layer) and negative (with lipopolysaccharide layer) bacteria is different from each other; as a result, in gram-negative bacteria, the lipopolysaccharide layer limits the passage and solubility of extract compounds in their cell membrane (Behbahani et al., 2019).

### 3.6. Antibacterial effect of *P. ferulacea* extract on microorganism morphology

**Figure 6:**
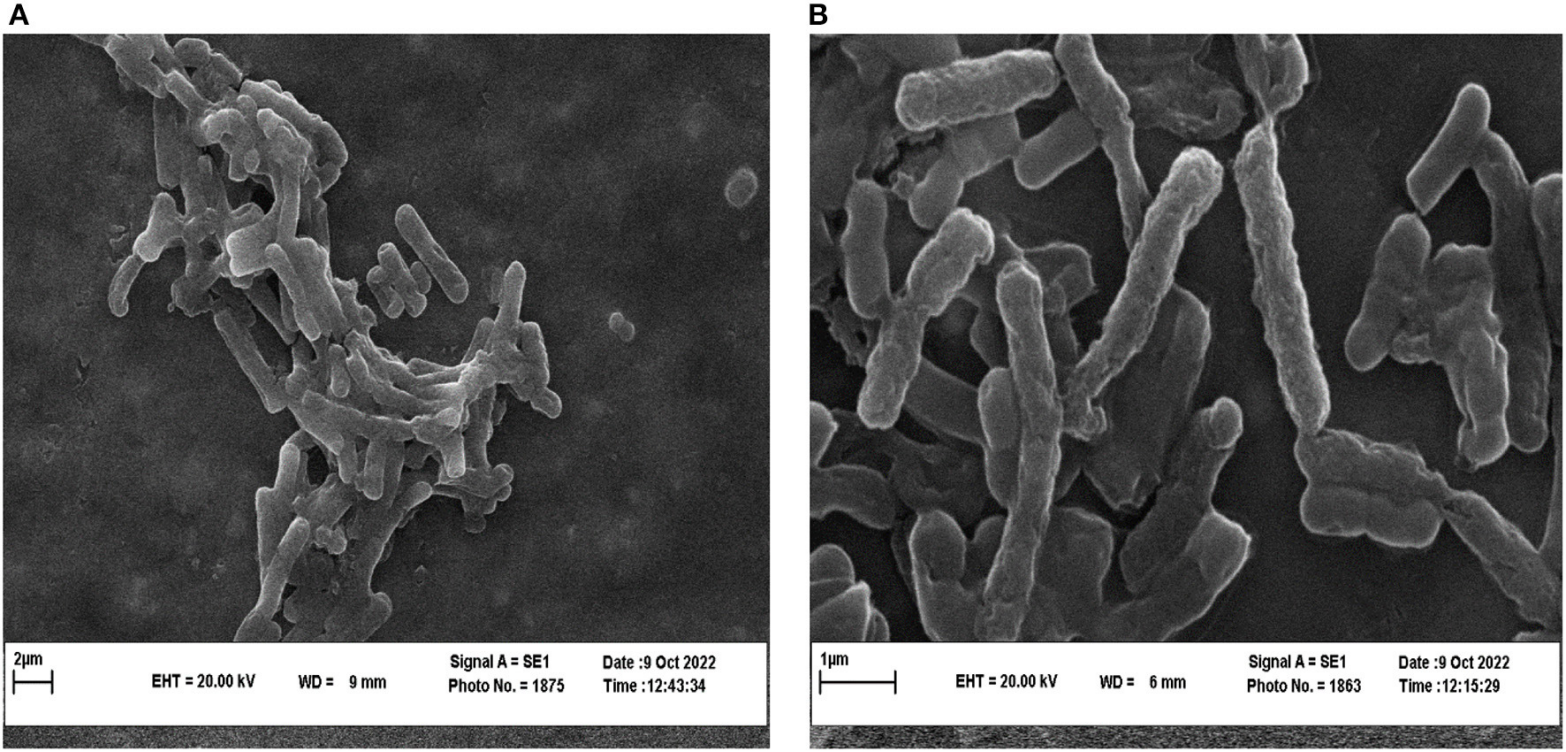
SEM images of *L. monocytogenes* control **(A)** and *L. monocytogenes* treated with extract **(B)**.

To investigate the effect of *P. ferulacea* extract on the structure of *L. monocytogenes* as the bacteria that showed the most sensitivity to the extract, SEM and CLSM images were prepared. SEM images of *L. monocytogenes* before and after treatment with *P. ferulacea* extract are shown in Figure 6. As can be seen from Figure 6, the structure of *L. monocytogenes* is naturally double coccobacillus and the treatment of bacteria with the extract caused changes in its structure. The extract caused cell wall wrinkling and indentation, cell folding, cell wall rupture, leakage of intracellular substances to the outside of the cell, and ultimately cell lysis. Behbahani et al. (2019) investigated the morphology of *L. innocua* and *E. coli* strains treated with *Cinnamomum zeylanicum* essential oil through SEM. Their results showed that this essential oil changed the shape and disordered the structure of bacteria (Alizadeh Behbahani et al., 2020). Li et al. (2021) investigated the effect of shikonin compound on *L. monocytogenes* ATCC 19,115 cells. They reported that in the untreated sample, biofilms of *L. monocytogenes* are thick, heterogeneous, and strongly accumulated, but, after treatment with shikonin, the integrity, three-dimensional structure, and the number of bacteria attached to the surface gradually decreased and single bacterial cells were observed (Li et al., 2021).

**Figure 7:**
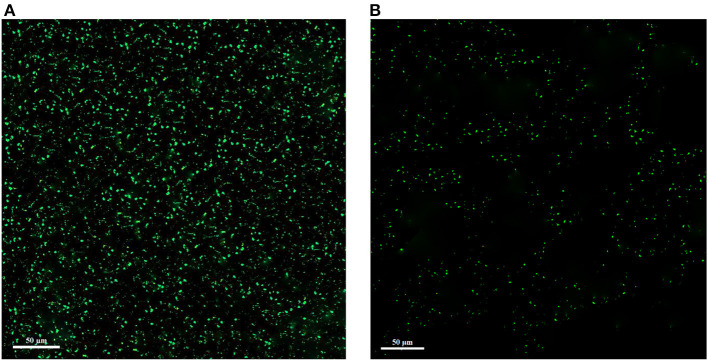
Confocal laser scanning microscopy (CLSM) images of *L. monocytogenes* control **(A)** and *L. monocytogenes* treated with extract **(B)**.

The corresponding CLSM images are shown in Figure 7. Since green color is used to stain alive cells, in the control sample without treatment with the extract, the cells are clearly green in the form of dots, but in the treated sample, the green dots are reduced. As a result, these images showed a significant reduction of living cells and the antimicrobial effect of the extract. In general, biofilm formation by *L. monocytogenes* is a defense mechanism against adverse antimicrobial conditions (high concentration of antimicrobial substances, sugar or salt, inappropriate temperature, and pH), which is the cause of disease and a threat to food and industry safety (Harvey et al., 2007).

### 3.7. Effect of *P. ferulacea* on the transcription of genes related to biofilm formation

**Figure 8:**
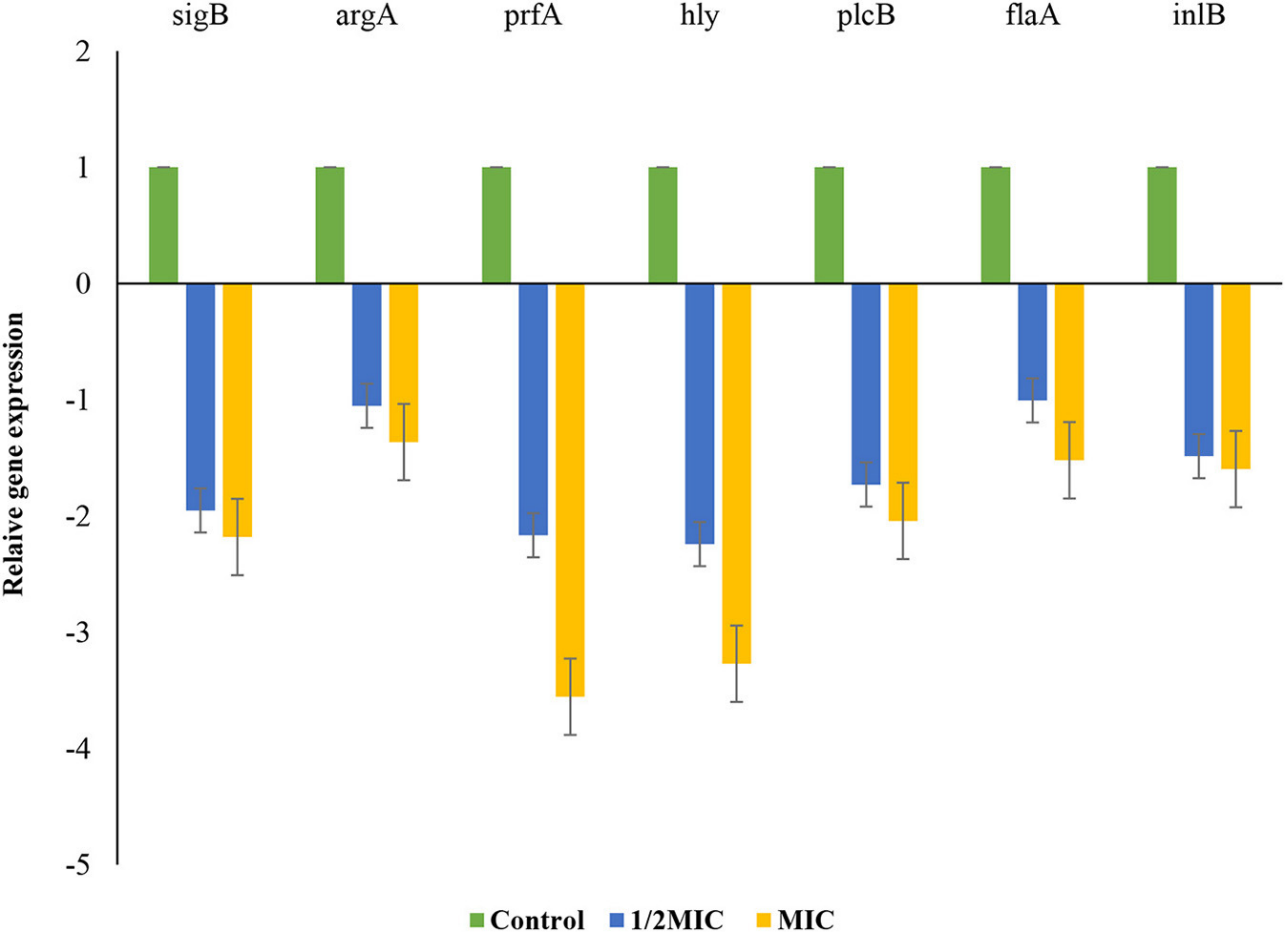
Effects of extract on the transcription of *L. monocytogenes* biofilm- and virulence-associated genes. Bars represent the standard deviation (*n* = 3).

*L. monocytogenes* in the late stage of growth with concentration of MIC and 1/2 MIC was investigated for its biofilm formation. The effect of *P. ferulacea* aqueous extracts on the transcription of genes related to biofilm formation, including quorum sensing gene (*agrA*), virulence factors (prfA), listeriolysin O gene (hly), stress response factor (sigB), phosphatidylcholine phospholipase-encoding gene (plcB), internalization protein regulatory gene (inlB), and flagella (flaA), is shown in Figure 8. In the sample treated with 1/2 of the extract, the expression of sigB, prfA, hly, and plcB genes decreased significantly, but at a higher concentration of the extract, the transcription of all genes except argA showed a significant decrease. sigB gene helps to regulate stress response genes so that *L. monocytogenes* survives in adverse environmental conditions (Moorhead et al., 2003). In this study, the extract caused a decrease in the transcription of the flagellum gene (flaA), which, through adhesion, plays an important role in *L. monocytogenes* motility and biofilm formation (Lemon et al., 2007; Miao et al., 2019). Adhesion and invasion processes are controlled by *plcB* and *inlB* genes, which play a role in directing bacteria to enter host cells, spread between cells, or escape from the innate immune system. Phospholipases, encoded by plcA and plcB, prevent *L. monocytogenes* from being entrapped within the internalization vacuole, thus allowing spread into the cytoplasm (Pizarro-Cerda and Cossart, 2018). Since *P. ferulacea* extract limited the transcription of hly and plcB in *L. monocytogenes*, it probably reduced the infection and biofilm formation of *L. monocytogenes* through the secretion of hemolysin and the control of phospholipase activity (Kim et al., 2021; Li et al., 2021).

## 4. Conclusion

Plants with medicinal and nutritional capabilities have always played a role in human life. Its medicinal properties are important for the treatment and prevention of many diseases, and it also improves the flavor of food. The *P. ferulacea* plant is found in the south-west of Iran, its aqueous extract was isolated, and its chemical characteristics showed that it contained catechin, rutin, p-coumaric acid, myricetin, caffeic acid, luteolin, and kaempferol. It also has TPC, TFC, and beta-carotene values of 202.04 ± 5.46 mg GAE/g DW, 1,909.46 ± 13 μg QE/g of DW, and 2.91 mg/100 g, respectively. By examining its antioxidant characteristics and comparing with other studies, it was determined that this plant has a good antioxidant power. Evaluation of its antimicrobial properties showed that it has a high antimicrobial power, especially against gram-positive bacteria, and *L. monocytogenes*, and *P. ferulacea* extract caused changes in its structural characteristics. This plant had a negative effect on the transcription of genes involved in the formation of *L. monocytogenes* biofilm. In general, this study shows that the *P. ferulacea* plant has significant antioxidant, anticancer, and antimicrobial properties, and it might be employed as a supplemental or alternative method to lessen infections brought on by *L. monocytogenes*. Animal studies of *P. ferulacea* extract on virulence factors of *Listeria* and the molecular mechanism *in vivo* should be explored in future studies.

## Data availability statement

The original contributions presented in the study are included in the article/supplementary material, further inquiries can be directed to the corresponding author.

## Author contributions

SJ: conceptualization, investigation, and writing—original draft. BA and MH: conceptualization, resources, supervision, methodology, and writing—reviewing and editing. AV and MN: investigation, methodology, and writing—reviewing and editing. All authors contributed to the article and approved the submitted version.
